# Crystal structure of 1,3-bis­{[4-(acetyl­sulfanyl)phenyl]ethynyl}azulene

**DOI:** 10.1107/S2056989016000323

**Published:** 2015-12-31

**Authors:** Sebastian Förster, Wilhelm Seichter, Edwin Weber

**Affiliations:** a Institut für Organische Chemie, TU Bergakademie Freiberg, Leipziger Strasse, 29, D-09596 Freiberg/Sachsen, Germany

**Keywords:** crystal structure, azulene, 1,3-disubstitution, C—H⋯O hydrogen bond, C—H⋯π inter­action

## Abstract

In the title compound, C_30_H_20_O_2_S_2_, the dihedral angles between the central azulene ring system (r.m.s. deviation = 0.039 Å) and the pendant benzene rings are 28.96 (7) and 55.15 (7)°. The dihedral angles between the benzene rings and their attached acetyl­sulfanyl groups are 59.60 (10) and 84.79 (10)°. The expected π–π stacking inter­actions are not observed in the crystal structure; instead, the packing features C—H⋯O hydrogen bonds, which link the mol­ecules into *C*(12) [010] chains, which are supported by weak C—H⋯π contacts.

## Related literature   

For background to this work, see: Wang *et al.* (2009 ▸); Puodziukynaite *et al.* (2014 ▸); Xia *et al.* (2014 ▸). For the synthesis and related structures, see: Förster *et al.* (2012 ▸, 2014 ▸).
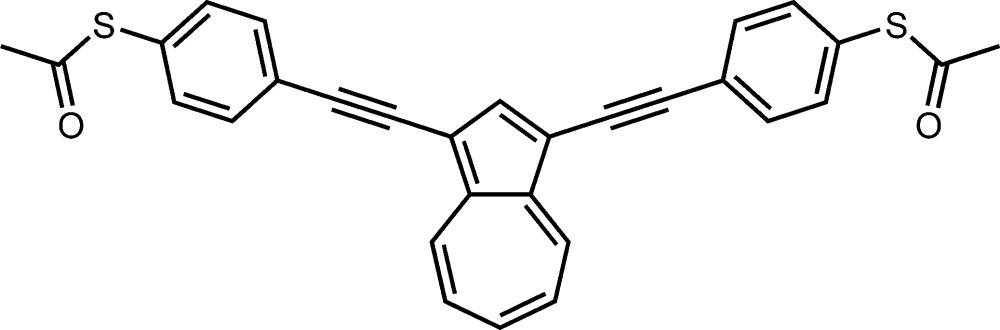


## Experimental   

### Crystal data  

C_30_H_20_O_2_S_2_
*M* *_r_* = 476.58
Monoclinic, 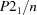

*a* = 13.7674 (3) Å
*b* = 8.9849 (2) Å
*c* = 19.7586 (4) Å
β = 104.022 (1)°
*V* = 2371.28 (9) Å^3^
*Z* = 4
Mo *K*α radiation
μ = 0.25 mm^−1^
*T* = 100 K
0.24 × 0.23 × 0.15 mm

### Data collection  

Bruker Kappa APEX CCD diffractometer
Absorption correction: multi-scan (*SADABS*; Bruker, 2008 ▸) *T*_min_ = 0.942, *T*_max_ = 0.963
36340 measured reflections
5898 independent reflections
4422 reflections with *I* > 2σ(*I*)
*R*_int_ = 0.039

### Refinement  

*R*[*F* ^2^ > 2σ(*F* ^2^)] = 0.046
*wR*(*F* ^2^) = 0.130
*S* = 1.03
5898 reflections
309 parameters
H-atom parameters constrained
Δρ_max_ = 0.44 e Å^−3^
Δρ_min_ = −0.27 e Å^−3^

### 

Data collection: *APEX2* (Bruker AXS); cell refinement: *SAINT* (Sheldrick, 2008 ▸); data reduction: *SAINT*; program(s) used to solve structure: *SHELXS97* (Sheldrick, 2008 ▸); program(s) used to refine structure: *SHELXL2015* (Sheldrick, 2015 ▸); molecular graphics: *ORTEP-3 for Windows* (Farrugia, 2012 ▸); software used to prepare material for publication: *SHELXTL* (Sheldrick, 2008 ▸).

## Supplementary Material

Crystal structure: contains datablock(s) I, publication_text. DOI: 10.1107/S2056989016000323/hb7546sup1.cif

Structure factors: contains datablock(s) I. DOI: 10.1107/S2056989016000323/hb7546Isup2.hkl

Supporting information file. DOI: 10.1107/S2056989016000323/hb7546Isup3.cml

**Fig. 1.**
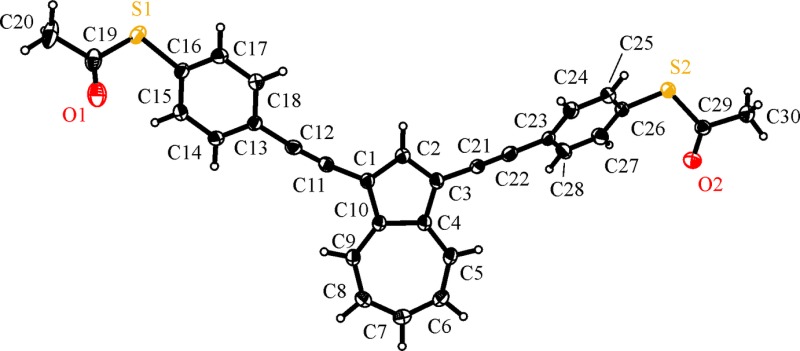
Ellipsoid plot.

. DOI: 10.1107/S2056989016000323/hb7546fig1.tif
Ellipsoid plot.

**Fig. 2.**
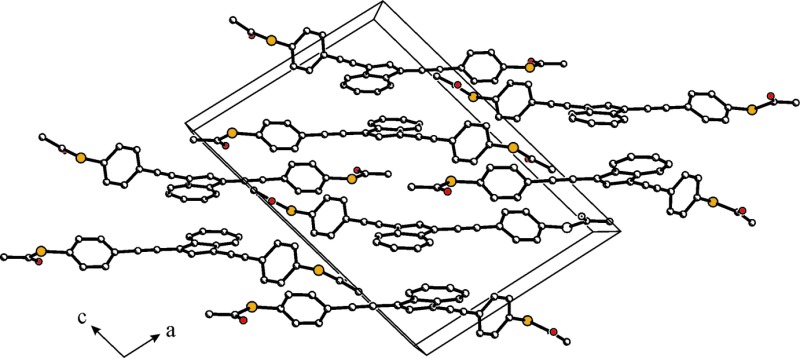
Packing diagram.

. DOI: 10.1107/S2056989016000323/hb7546fig2.tif
Packing diagram.

CCDC reference: 1445850

Additional supporting information:  crystallographic information; 3D view; checkCIF report

## Figures and Tables

**Table 1:** Hydrogen-bond geometry (Å, °) *Cg*1 is the mid-point of the C11—C12 bond and *Cg*2 is the centroid of the C1–C4/C10 ring.

*D*—H⋯*A*	*D*—H	H⋯*A*	*D*⋯*A*	*D*—H⋯*A*
C2—H2⋯O1^i^	0.95	2.40	3.285 (3)	155
C17—H1⋯*Cg*1	0.95	2.69	3.612 (3)	165
C20—H20*A*⋯*Cg*2^ii^	0.98	2.89	3.835 (2)	162

Symmetry code: (i) 
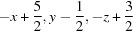
; (ii) 
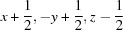
.

## supplementary crystallographic information

### S1. Comment

Azulene derivatives offer a number of inter­esting applications especially in the field of molecular electronics (Wang *et al.*, 2009; Puodziukynaite *et al.*, 2014). It ties up with the fact that the non-alternating azulene possesses remarkable electronic and optical properties (Xia *et al.*, 2014). Although the title compound, C_30_H_20_O_2_S_2_, (I), is fully conjugated, no flat molecular structure can be observed. Both phenyl rings, fig 1, are rotated out of the plane containing the azulene core [*phenyl*(C13—C18) 29.0°, *phenyl*(C23—C28) 55.2°]. The C—S—C angle of the acetyl protected thiol is slightly smaller compared to that found in an analogous compound,1,3-bis­[4-(*tert*-butyl­sulfanyl)phenyl­ethynyl]azulene, featuring a *tert*-butyl protection group at the sulfur atom (Förster *et al.*, 2012). Unlike the previous case, no π···π inter­actions are present in the title compound. In all probability, this uncommon phenomenon within the substance class of azulenes (Förster *et al.*, 2014) is related to the non-planar molecular structure and may be caused from packing effects. However, the crystal structure is based on C—H···O hydrogen bonds [C2—-H2···O1 (2.5-x, -0.5+y, 1.5-z; 2.40 Å, 155.0°)] and C—H···π inter­actions [C17—H17···Cg(1) 2.69 Å, 164.9°; C20—H20A···Cg(2) 2.89 Å, 159.0°].

### S2.1. Synthesis and crystallization

The synthesis of the title compound, (I), has already been described (Förster *et al.* 2012). The crystals were grown from toluene solution by slow evaporation.

### S2.2. Refinement

Crystal data, data collection and structure refinement details are summarized in Table 1. The hydrogen atoms attached to C were fixed geometrically and treated as riding atoms, with d(C—H) = 0.93 and Uiso(H) = 1.2 Ueq(C) for aromatic and Uiso(H) = 1.5Ueq(C) for methyl groups.

### Crystal data

**Table:**

C_30_H_20_O_2_S_2_	*F*(000) = 992
*M**_r_* = 476.58	*D*_x_ = 1.335 Mg m^−^^3^
Monoclinic, *P*2_1_/*n*	Mo *K*α radiation, λ = 0.71073 Å
*a* = 13.7674 (3) Å	Cell parameters from 8057 reflections
*b* = 8.9849 (2) Å	θ = 2.5–28.0°
*c* = 19.7586 (4) Å	µ = 0.25 mm^−^^1^
β = 104.022 (1)°	*T* = 100 K
*V* = 2371.28 (9) Å^3^	Irregular, green
*Z* = 4	0.24 × 0.23 × 0.15 mm

### Data collection

**Table:**

Bruker Kappa APEX CCD diffractometer	4422 reflections with *I* > 2σ(*I*)
phi and ω scans	*R*_int_ = 0.039
Absorption correction: multi-scan (*SADABS*; Bruker, 2008)	θ_max_ = 28.3°, θ_min_ = 2.5°
*T*_min_ = 0.942, *T*_max_ = 0.963	*h* = −18→18
36340 measured reflections	*k* = −12→11
5898 independent reflections	*l* = −26→26

### Refinement

**Table:**

Refinement on *F*^2^	0 restraints
Least-squares matrix: full	Hydrogen site location: inferred from neighbouring sites
*R*[*F*^2^ > 2σ(*F*^2^)] = 0.046	H-atom parameters constrained
*wR*(*F*^2^) = 0.130	*w* = 1/[σ^2^(*F*_o_^2^) + (0.0658*P*)^2^ + 1.347*P*] where *P* = (*F*_o_^2^ + 2*F*_c_^2^)/3
*S* = 1.03	(Δ/σ)_max_ = 0.001
5898 reflections	Δρ_max_ = 0.44 e Å^−^^3^
309 parameters	Δρ_min_ = −0.27 e Å^−^^3^

### Special details

**Table:**

Geometry. All e.s.d.'s (except the e.s.d. in the dihedral angle between two l.s. planes) are estimated using the full covariance matrix. The cell e.s.d.'s are taken into account individually in the estimation of e.s.d.'s in distances, angles and torsion angles; correlations between e.s.d.'s in cell parameters are only used when they are defined by crystal symmetry. An approximate (isotropic) treatment of cell e.s.d.'s is used for estimating e.s.d.'s involving l.s. planes.

### Fractional atomic coordinates and isotropic or equivalent isotropic displacement parameters (Å^2^)

**Table:**

	*x*	*y*	*z*	*U*_iso_*/*U*_eq_	
S1	1.37569 (4)	0.11544 (6)	0.58441 (3)	0.03405 (15)	
S2	0.55913 (4)	−0.42293 (6)	1.10153 (3)	0.03144 (14)	
O1	1.43557 (12)	0.39449 (19)	0.60090 (9)	0.0418 (4)	
O2	0.52513 (12)	−0.17076 (16)	1.16136 (8)	0.0361 (4)	
C1	0.93413 (13)	0.2985 (2)	0.81129 (9)	0.0229 (4)	
C2	0.91025 (14)	0.1731 (2)	0.84616 (10)	0.0248 (4)	
H2	0.9425	0.0791	0.8485	0.030*	
C3	0.83172 (14)	0.2075 (2)	0.87699 (10)	0.0235 (4)	
C4	0.80547 (13)	0.3585 (2)	0.86356 (9)	0.0224 (4)	
C5	0.73471 (14)	0.4346 (2)	0.88954 (10)	0.0258 (4)	
H5	0.7021	0.3782	0.9182	0.031*	
C6	0.70505 (15)	0.5828 (2)	0.87924 (11)	0.0286 (4)	
H6	0.6574	0.6156	0.9036	0.034*	
C7	0.73635 (14)	0.6887 (2)	0.83792 (10)	0.0281 (4)	
H7	0.7048	0.7831	0.8366	0.034*	
C8	0.80679 (15)	0.6777 (2)	0.79820 (10)	0.0279 (4)	
H8	0.8147	0.7643	0.7725	0.033*	
C9	0.86748 (14)	0.5575 (2)	0.79079 (10)	0.0244 (4)	
H9	0.9129	0.5744	0.7623	0.029*	
C10	0.86986 (13)	0.4173 (2)	0.81964 (9)	0.0217 (4)	
C11	1.01077 (14)	0.2995 (2)	0.77358 (10)	0.0235 (4)	
C12	1.07506 (14)	0.2876 (2)	0.74377 (10)	0.0252 (4)	
C13	1.15323 (13)	0.2606 (2)	0.70827 (10)	0.0222 (4)	
C14	1.15694 (14)	0.3358 (2)	0.64730 (10)	0.0261 (4)	
H14	1.1120	0.4155	0.6312	0.031*	
C15	1.22577 (14)	0.2948 (2)	0.61000 (10)	0.0272 (4)	
H15	1.2263	0.3440	0.5675	0.033*	
C16	1.29404 (13)	0.1821 (2)	0.63447 (10)	0.0242 (4)	
C17	1.29573 (14)	0.1142 (2)	0.69783 (10)	0.0270 (4)	
H17	1.3454	0.0420	0.7163	0.032*	
C18	1.22522 (14)	0.1517 (2)	0.73400 (10)	0.0269 (4)	
H18	1.2256	0.1031	0.7768	0.032*	
C19	1.44675 (15)	0.2760 (3)	0.57564 (11)	0.0328 (5)	
C20	1.52157 (17)	0.2459 (3)	0.53316 (12)	0.0483 (7)	
H20A	1.4865	0.2112	0.4866	0.072*	
H20B	1.5688	0.1692	0.5562	0.072*	
H20C	1.5582	0.3375	0.5289	0.072*	
C21	0.78423 (15)	0.1054 (2)	0.91530 (9)	0.0243 (4)	
C22	0.74797 (15)	0.0199 (2)	0.94649 (10)	0.0275 (4)	
C23	0.70236 (14)	−0.0862 (2)	0.98362 (10)	0.0246 (4)	
C24	0.76034 (15)	−0.1665 (2)	1.03950 (10)	0.0284 (4)	
H24	0.8306	−0.1504	1.0535	0.034*	
C25	0.71595 (15)	−0.2691 (2)	1.07442 (11)	0.0289 (4)	
H25	0.7557	−0.3248	1.1119	0.035*	
C26	0.61271 (15)	−0.2908 (2)	1.05464 (10)	0.0268 (4)	
C27	0.55486 (15)	−0.2111 (2)	1.00026 (11)	0.0318 (5)	
H27	0.4845	−0.2261	0.9870	0.038*	
C28	0.59931 (15)	−0.1085 (2)	0.96468 (11)	0.0308 (4)	
H28	0.5592	−0.0532	0.9272	0.037*	
C29	0.52713 (14)	−0.3041 (2)	1.16535 (10)	0.0261 (4)	
C30	0.50479 (17)	−0.3905 (2)	1.22472 (11)	0.0341 (5)	
H30A	0.5636	−0.3895	1.2643	0.051*	
H30B	0.4883	−0.4935	1.2100	0.051*	
H30C	0.4479	−0.3451	1.2387	0.051*	

### Atomic displacement parameters (Å^2^)

**Table:**

	*U*^11^	*U*^22^	*U*^33^	*U*^12^	*U*^13^	*U*^23^
S1	0.0321 (3)	0.0331 (3)	0.0435 (3)	0.0021 (2)	0.0220 (2)	−0.0023 (2)
S2	0.0425 (3)	0.0203 (2)	0.0379 (3)	−0.0023 (2)	0.0221 (2)	0.0025 (2)
O1	0.0341 (8)	0.0477 (10)	0.0446 (9)	−0.0138 (7)	0.0117 (7)	−0.0013 (8)
O2	0.0478 (9)	0.0248 (8)	0.0381 (8)	0.0010 (7)	0.0152 (7)	0.0001 (6)
C1	0.0234 (9)	0.0222 (9)	0.0244 (9)	−0.0025 (7)	0.0085 (7)	−0.0004 (7)
C2	0.0276 (9)	0.0225 (9)	0.0260 (9)	−0.0003 (7)	0.0097 (7)	−0.0001 (8)
C3	0.0246 (9)	0.0238 (9)	0.0233 (9)	−0.0034 (7)	0.0083 (7)	0.0004 (7)
C4	0.0224 (8)	0.0239 (9)	0.0217 (9)	−0.0023 (7)	0.0069 (7)	−0.0007 (7)
C5	0.0232 (9)	0.0301 (10)	0.0266 (9)	−0.0025 (8)	0.0108 (7)	−0.0010 (8)
C6	0.0259 (9)	0.0312 (11)	0.0309 (10)	0.0015 (8)	0.0114 (8)	−0.0043 (8)
C7	0.0273 (9)	0.0266 (10)	0.0293 (10)	0.0039 (8)	0.0048 (8)	−0.0027 (8)
C8	0.0314 (10)	0.0241 (10)	0.0277 (10)	0.0006 (8)	0.0062 (8)	0.0041 (8)
C9	0.0259 (9)	0.0261 (10)	0.0224 (9)	−0.0024 (7)	0.0082 (7)	0.0002 (7)
C10	0.0208 (8)	0.0237 (9)	0.0213 (8)	−0.0023 (7)	0.0067 (7)	−0.0012 (7)
C11	0.0265 (9)	0.0180 (9)	0.0269 (9)	−0.0045 (7)	0.0083 (7)	−0.0018 (7)
C12	0.0272 (9)	0.0179 (9)	0.0309 (10)	−0.0011 (7)	0.0078 (8)	−0.0020 (7)
C13	0.0222 (8)	0.0193 (9)	0.0267 (9)	−0.0021 (7)	0.0093 (7)	−0.0034 (7)
C14	0.0234 (9)	0.0230 (10)	0.0331 (10)	0.0033 (7)	0.0093 (8)	0.0033 (8)
C15	0.0283 (9)	0.0271 (10)	0.0284 (10)	0.0010 (8)	0.0111 (8)	0.0059 (8)
C16	0.0217 (8)	0.0241 (10)	0.0293 (9)	−0.0010 (7)	0.0113 (7)	−0.0016 (8)
C17	0.0244 (9)	0.0240 (10)	0.0335 (10)	0.0033 (7)	0.0085 (8)	0.0027 (8)
C18	0.0293 (10)	0.0256 (10)	0.0279 (10)	0.0009 (8)	0.0108 (8)	0.0023 (8)
C19	0.0232 (9)	0.0482 (14)	0.0265 (10)	−0.0047 (9)	0.0051 (8)	0.0029 (9)
C20	0.0271 (11)	0.084 (2)	0.0374 (12)	−0.0038 (12)	0.0152 (9)	0.0081 (13)
C21	0.0329 (10)	0.0213 (9)	0.0204 (9)	0.0035 (8)	0.0095 (7)	−0.0006 (7)
C22	0.0301 (10)	0.0246 (10)	0.0304 (10)	0.0002 (8)	0.0126 (8)	−0.0050 (8)
C23	0.0321 (10)	0.0181 (9)	0.0284 (9)	−0.0015 (7)	0.0169 (8)	−0.0030 (7)
C24	0.0273 (9)	0.0285 (10)	0.0318 (10)	−0.0006 (8)	0.0115 (8)	0.0010 (8)
C25	0.0325 (10)	0.0262 (10)	0.0304 (10)	0.0028 (8)	0.0124 (8)	0.0046 (8)
C26	0.0357 (10)	0.0184 (9)	0.0314 (10)	−0.0013 (8)	0.0181 (8)	0.0002 (8)
C27	0.0253 (9)	0.0294 (11)	0.0424 (12)	−0.0021 (8)	0.0117 (8)	0.0057 (9)
C28	0.0309 (10)	0.0271 (10)	0.0358 (11)	0.0019 (8)	0.0110 (8)	0.0096 (9)
C29	0.0248 (9)	0.0233 (10)	0.0311 (10)	−0.0001 (7)	0.0085 (8)	0.0012 (8)
C30	0.0416 (12)	0.0305 (11)	0.0355 (11)	0.0012 (9)	0.0195 (9)	0.0028 (9)

### Geometric parameters (Å, º)

**Table:**

S1—C16	1.7716 (19)	C14—C15	1.384 (3)
S1—C19	1.775 (2)	C14—H14	0.9500
S2—C26	1.7727 (19)	C15—C16	1.387 (3)
S2—C29	1.787 (2)	C15—H15	0.9500
O1—C19	1.201 (3)	C16—C17	1.387 (3)
O2—C29	1.201 (2)	C17—C18	1.380 (3)
C1—C2	1.401 (3)	C17—H17	0.9500
C1—C10	1.421 (3)	C18—H18	0.9500
C1—C11	1.432 (2)	C19—C20	1.503 (3)
C2—C3	1.398 (3)	C20—H20A	0.9800
C2—H2	0.9500	C20—H20B	0.9800
C3—C4	1.413 (3)	C20—H20C	0.9800
C3—C21	1.443 (3)	C21—C22	1.170 (3)
C4—C5	1.386 (3)	C22—C23	1.437 (3)
C4—C10	1.480 (2)	C23—C28	1.391 (3)
C5—C6	1.393 (3)	C23—C24	1.396 (3)
C5—H5	0.9500	C24—C25	1.380 (3)
C6—C7	1.388 (3)	C24—H24	0.9500
C6—H6	0.9500	C25—C26	1.394 (3)
C7—C8	1.391 (3)	C25—H25	0.9500
C7—H7	0.9500	C26—C27	1.374 (3)
C8—C9	1.394 (3)	C27—C28	1.388 (3)
C8—H8	0.9500	C27—H27	0.9500
C9—C10	1.380 (3)	C28—H28	0.9500
C9—H9	0.9500	C29—C30	1.500 (3)
C11—C12	1.181 (3)	C30—H30A	0.9800
C12—C13	1.440 (3)	C30—H30B	0.9800
C13—C14	1.393 (3)	C30—H30C	0.9800
C13—C18	1.398 (3)		
			
C16—S1—C19	102.66 (10)	C17—C16—S1	118.76 (15)
C26—S2—C29	100.01 (9)	C18—C17—C16	120.01 (18)
C2—C1—C10	108.67 (16)	C18—C17—H17	120.0
C2—C1—C11	123.48 (17)	C16—C17—H17	120.0
C10—C1—C11	127.85 (17)	C17—C18—C13	120.56 (18)
C3—C2—C1	109.57 (17)	C17—C18—H18	119.7
C3—C2—H2	125.2	C13—C18—H18	119.7
C1—C2—H2	125.2	O1—C19—C20	124.5 (2)
C2—C3—C4	108.68 (16)	O1—C19—S1	123.40 (16)
C2—C3—C21	125.77 (18)	C20—C19—S1	112.13 (18)
C4—C3—C21	125.55 (17)	C19—C20—H20A	109.5
C5—C4—C3	125.09 (17)	C19—C20—H20B	109.5
C5—C4—C10	128.00 (18)	H20A—C20—H20B	109.5
C3—C4—C10	106.88 (16)	C19—C20—H20C	109.5
C4—C5—C6	128.65 (18)	H20A—C20—H20C	109.5
C4—C5—H5	115.7	H20B—C20—H20C	109.5
C6—C5—H5	115.7	C22—C21—C3	178.1 (2)
C7—C6—C5	128.24 (18)	C21—C22—C23	178.9 (2)
C7—C6—H6	115.9	C28—C23—C24	119.25 (17)
C5—C6—H6	115.9	C28—C23—C22	120.10 (18)
C6—C7—C8	129.88 (19)	C24—C23—C22	120.64 (18)
C6—C7—H7	115.1	C25—C24—C23	120.20 (18)
C8—C7—H7	115.1	C25—C24—H24	119.9
C7—C8—C9	129.18 (19)	C23—C24—H24	119.9
C7—C8—H8	115.4	C24—C25—C26	119.92 (19)
C9—C8—H8	115.4	C24—C25—H25	120.0
C10—C9—C8	128.20 (18)	C26—C25—H25	120.0
C10—C9—H9	115.9	C27—C26—C25	120.30 (18)
C8—C9—H9	115.9	C27—C26—S2	121.48 (15)
C9—C10—C1	126.22 (17)	C25—C26—S2	118.21 (15)
C9—C10—C4	127.60 (17)	C26—C27—C28	119.96 (19)
C1—C10—C4	106.17 (16)	C26—C27—H27	120.0
C12—C11—C1	174.3 (2)	C28—C27—H27	120.0
C11—C12—C13	175.5 (2)	C27—C28—C23	120.36 (19)
C14—C13—C18	118.85 (17)	C27—C28—H28	119.8
C14—C13—C12	121.86 (17)	C23—C28—H28	119.8
C18—C13—C12	119.23 (17)	O2—C29—C30	124.21 (19)
C15—C14—C13	120.34 (18)	O2—C29—S2	123.75 (16)
C15—C14—H14	119.8	C30—C29—S2	112.03 (14)
C13—C14—H14	119.8	C29—C30—H30A	109.5
C14—C15—C16	120.16 (17)	C29—C30—H30B	109.5
C14—C15—H15	119.9	H30A—C30—H30B	109.5
C16—C15—H15	119.9	C29—C30—H30C	109.5
C15—C16—C17	119.82 (17)	H30A—C30—H30C	109.5
C15—C16—S1	121.35 (15)	H30B—C30—H30C	109.5
			
C10—C1—C2—C3	0.1 (2)	C13—C14—C15—C16	−2.4 (3)
C11—C1—C2—C3	−179.26 (17)	C14—C15—C16—C17	−2.5 (3)
C1—C2—C3—C4	−1.4 (2)	C14—C15—C16—S1	174.31 (15)
C1—C2—C3—C21	177.71 (18)	C19—S1—C16—C15	61.42 (18)
C2—C3—C4—C5	−175.89 (18)	C19—S1—C16—C17	−121.78 (17)
C21—C3—C4—C5	5.0 (3)	C15—C16—C17—C18	4.4 (3)
C2—C3—C4—C10	2.2 (2)	S1—C16—C17—C18	−172.40 (15)
C21—C3—C4—C10	−176.99 (17)	C16—C17—C18—C13	−1.6 (3)
C3—C4—C5—C6	179.6 (2)	C14—C13—C18—C17	−3.1 (3)
C10—C4—C5—C6	1.9 (3)	C12—C13—C18—C17	174.31 (18)
C4—C5—C6—C7	3.0 (4)	C16—S1—C19—O1	0.8 (2)
C5—C6—C7—C8	−2.4 (4)	C16—S1—C19—C20	−179.13 (15)
C6—C7—C8—C9	−2.1 (4)	C28—C23—C24—C25	1.4 (3)
C7—C8—C9—C10	2.4 (4)	C22—C23—C24—C25	−179.43 (18)
C8—C9—C10—C1	−179.16 (19)	C23—C24—C25—C26	−1.1 (3)
C8—C9—C10—C4	2.5 (3)	C24—C25—C26—C27	0.3 (3)
C2—C1—C10—C9	−177.39 (18)	C24—C25—C26—S2	−179.55 (15)
C11—C1—C10—C9	1.9 (3)	C29—S2—C26—C27	−86.86 (18)
C2—C1—C10—C4	1.2 (2)	C29—S2—C26—C25	92.98 (17)
C11—C1—C10—C4	−179.46 (18)	C25—C26—C27—C28	0.1 (3)
C5—C4—C10—C9	−5.5 (3)	S2—C26—C27—C28	179.98 (16)
C3—C4—C10—C9	176.53 (18)	C26—C27—C28—C23	0.2 (3)
C5—C4—C10—C1	175.89 (19)	C24—C23—C28—C27	−1.0 (3)
C3—C4—C10—C1	−2.1 (2)	C22—C23—C28—C27	179.86 (19)
C18—C13—C14—C15	5.1 (3)	C26—S2—C29—O2	14.4 (2)
C12—C13—C14—C15	−172.25 (18)	C26—S2—C29—C30	−164.72 (15)

### Hydrogen-bond geometry (Å, º)

Cg1 is the mid-point of C11—C12 and Cg2 is the centroid of the C1–C4/C10 ring.

**Table:**

*D*—H···*A*	*D*—H	H···*A*	*D*···*A*	*D*—H···*A*
C2—H2···O1^i^	0.95	2.40	3.285 (3)	155
C17—H1···*Cg*1	0.95	2.69	3.612 (3)	165
C20—H20*A*···*Cg*2^ii^	0.98	2.89	3.835 (2)	162

Symmetry codes: (i) −*x*+5/2, *y*−1/2, −*z*+3/2; (ii) *x*+1/2, −*y*+1/2, *z*−1/2.
