# Supplementary figures and images for: Crystal structure of 1,3-bis­{[4-(acetyl­sulfanyl)phenyl]ethynyl}azulene
Source: Acta Crystallogr E Crystallogr Commun. 2015 Dec 31;71(Pt 12):o1099–100. doi: 10.1107/S2056989016000323 (PMC4719999; doi:10.1107/S2056989016000323)

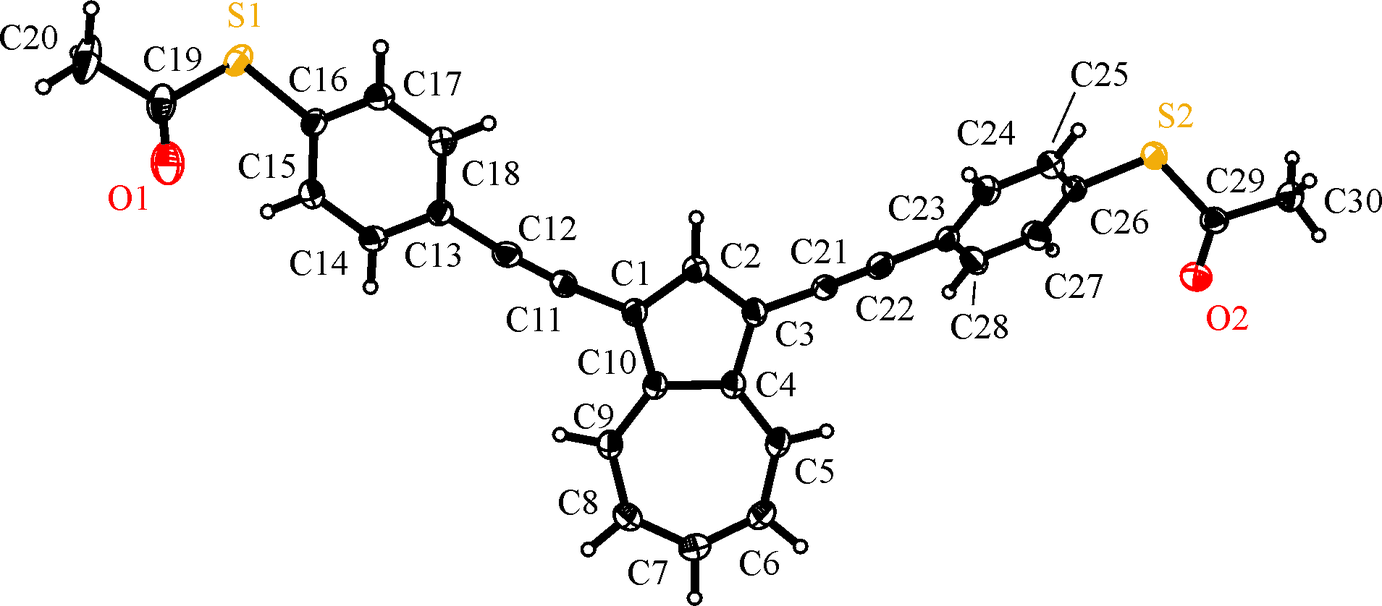

Supplement: Supplementary file 4 [file e-71-o1099-fig1.tif]

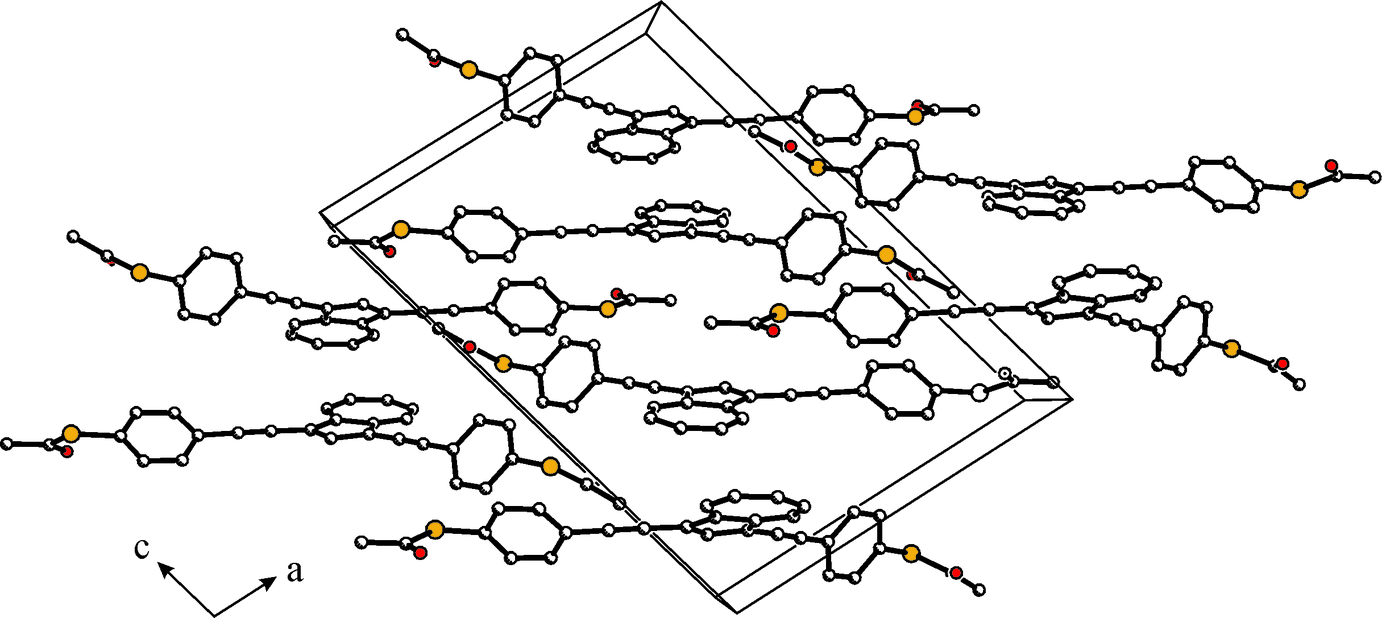

Supplement: Supplementary file 5 [file e-71-o1099-fig2.tif]
